# Altered NGF and GDNF levels reveal neuroimmune dysregulation in COVID-19 patients

**DOI:** 10.1038/s41598-026-40236-9

**Published:** 2026-02-19

**Authors:** Lale Saka Baraz, Evrim Ataca, Nur Duzen Oflas, Seyma Canavar Kosali, Busra Usta, Alihan Oral, Mustafa Cihangiroglu, Metin Ozgen, Demet Yalcin Kehribar

**Affiliations:** 1 Department of Internal Medicine, Manisa City Hospital, Manisa, Türkiye Turkey; 2 Faculty of Medicine, Department of Internal Medicine, Dokuz Eylül University, Izmir, Turkey; 3 Faculty of Medicine, Department of Internal Medicine, Yıl University, Van, Turkey; 4 Faculty of Medicine, Department of Microbiology, Samsun University, Samsun, Turkey; 5 Faculty of Medicine, Department of Internal Medicine, Biruni University, Istanbul, Turkey; 6 Department of Infectious Disease, State Hospital, Suluova, Turkey; 7 Medical Point Hospital, Department of Rheumatology, Izmir Ekonomi University, Izmir, Turkey; 8 Institute of Oncology, Dokuz Eylul University, Izmir, Turkey; 9 Department of Internal Medicine, İzmir Tepecik Education and Research Hospital, Health Science University, Izmir, Turkey

**Keywords:** COVID-19, NGF, GDNF, Neurotrophins, Inflammation, Biomarkers, Biomarkers, Diseases, Immunology, Neurology, Neuroscience

## Abstract

Neurotrophins such as nerve growth factor (NGF) and glial cell line-derived neurotrophic factor (GDNF) are crucial for neuronal maintenance and immune regulation. However, their dynamics during coronavirus disease 2019 (COVID-19) remain unclear. In this prospective study, 30 hospitalized patients with PCR-confirmed COVID-19 were evaluated longitudinally. Serum NGF, GDNF, and conventional inflammatory markers (CRP, ESR, fibrinogen, ferritin, D-dimer, LDH, hematological counts) were measured on Day 1, Day 4, and at discharge. A control group of 37 healthy individuals was included for cross-sectional comparison. Both NGF and GDNF levels were significantly lower in COVID-19 patients at admission compared with healthy individuals. NGF showed a modest early decline from Day 1 to Day 4, followed by partial recovery at discharge, whereas GDNF remained stable throughout hospitalization. Inflammatory markers demonstrated expected clinical trajectories: CRP, ESR, LDH, and fibrinogen decreased during recovery, while WBC, neutrophils, and platelets increased. Ferritin and D-dimer showed no meaningful temporal changes. NGF appears to reflect acute neuroimmune activation in COVID-19 and may serve as a dynamic biomarker of early inflammatory resolution. Conversely, GDNF remained persistently suppressed, suggesting a distinct role in chronic neuroimmune regulation. These findings highlight NGF and GDNF as potential targets for monitoring and modulating neuroimmune responses in COVID-19 and other inflammation-driven conditions.

## Introduction

Nerve growth factor (NGF) and glial cell line-derived neurotrophic factor (GDNF) are neurotrophins, having vital roles in survival, differentiation, and repair of neurons. ^1^ Emerging evidence demonstrates that these neurotrophins also have immunomodulatory functions, capable of influencing cytokine release, immune cell behavior, and tissue remodeling in inflammatory contexts. ^2^

Altered serum NGF and GDNF levels have been reported in autoimmune and autoinflammatory diseases such as Behçet’s disease, suggesting a role in inflammatory pathogenesis. ^3^ In respiratory diseases like asthma, COPD, and pulmonary fibrosis, NGF expression is elevated in pulmonary tissues, indicating potential involvement in airway remodeling and chronic inflammation.^4^NGF levels are also increased during acute exacerbations of asthma^5^and in allergic conjunctivitis^6^, highlighting its participation in mucosal inflammation.

In children with H1N1 influenza, elevated NGF and interleukin levels have been linked to disease severity, underscoring the broader neuroimmune relevance of NGF in viral infections. ^7^ Despite these findings, the role of neurotrophins in the pathophysiology of COVID-19 remains poorly understood. A pilot study suggests alterations in NGF and BDNF levels in adolescents with long COVID, but comprehensive longitudinal data are lacking. ^8^

Viral infections such as respiratory syncytial virus and HSV-1 are known to disrupt neurotrophin signaling, leading to enhanced epithelial survival or neuroinflammatory responses. ^9^ Moreover, in inflammatory states more broadly, GDNF and related growth factor ligands (GFLs) are upregulated in immune-related tissues and can influence cytokine production, including IL-1β and IL-6 suggesting a broader role for neurotrophins in orchestrating inflammation.^10^.

COVID-19 severity has been closely associated with elevated inflammatory and hematological markers such as CRP, ferritin, LDH, D-dimer, and lymphopenia, which reflect underlying immune dysregulation, understanding how neurotrophins interact with these markers may offer novel insights into disease pathophysiology. ^11–13^

This study investigates temporal changes in serum NGF and GDNF levels in hospitalized COVID-19 patients and their relationship to standard inflammatory markers. By including comparisons with healthy controls, we aim to clarify neuroimmune dynamics and assess the utility of neurotrophins as biomarkers or therapeutic targets in COVID-19.

## Materials and methods

We enrolled 30 hospitalized adult patients who tested positive for COVID-19 via RT-PCR and had findings consistent with COVID-19 pneumonia on chest CT imaging. All patients were over 18 years of age and required hospitalization for COVID-19 pneumonia, consistent with moderate disease severity according to WHO clinical progression criteria^14^. COVID-19 patients did not have known acute or chronic inflammatory, infectious, neurological or autoimmune diseases prior to infection. The control group of 37 healthy volunteers were included for cross-sectional comparison. The healthy controls were over 18 years of age and had no known acute or chronic inflammatory, infectious, neurological or autoimmune diseases. The study was conducted in accordance with the Declaration of Helsinki and received approval from the Van Yüzüncü Yıl University Non-Interventional Clinical Research Ethics Committee (Decision No: 2025/05 − 01, dated 16 May 2025). Written informed consent was obtained from all participants prior to inclusion.

### Study design and sample collection

Peripheral venous blood samples were obtained from each COVID-19 patient at three predefined time points during hospitalization. The first sampling was performed on the day of hospital admission and confirmation of COVID-19 diagnosis (Day 1). The second sample was collected on the fourth day of hospitalization (Day 4), and the third sampling was carried out on the day of discharge, which occurred between Day 7 and Day 14 depending on the patient’s clinical recovery.

The following laboratory parameters were measured from each blood sample:

White blood cell count (WBC), lymphocyte count, neutrophil count, platelet count, lactate dehydrogenase (LDH), fibrinogen, ferritin, erythrocyte sedimentation rate (ESR), C-reactive protein (CRP), D-dimer, nerve growth factor (NGF), and glial cell line-derived neurotrophic factor (GDNF).

Blood samples from healthy individuals were collected at a single time point, and serum NGF and GDNF levels were measured using the same procedures and under the same laboratory conditions as for the patient group.

Detailed clinical outcome data (e.g., ICU admission, oxygen requirement, or length of hospitalization), disease severity classification, and treatment information (such as corticosteroid or immunomodulatory therapy) were not systematically recorded for all patients. Therefore, analyses linking neurotrophin levels to clinical outcomes or treatment effects could not be performed.

### Biomarker assays

NGF concentrations were measured using a commercially available Human NGF ELISA Kit (AFG Bioscience, Cat No. EK713140, Northbrook, Illinois, USA). Measurements were performed using an automatic microplate photometer. NGF levels were determined by comparing sample absorbance values to a standard curve. The assay had a sensitivity of 0.2 ng/mL and a working range of 1.56–100 ng/mL. The inter-assay and intra-assay coefficients of variation were < 10% and < 8%, respectively. Samples exceeding the upper detection limit were appropriately diluted and measured in duplicate.

GDNF concentrations were determined using a Human GDNF ELISA Kit (AFG Bioscience, Cat No. EK710976, Northbrook, Illinois, USA), following the manufacturer’s instructions. The assay sensitivity was 8 pg/mL, with a detection range of 40–2000 pg/mL. The inter-assay and intra-assay coefficients of variation were < 10% and < 8%, respectively. Samples with concentrations above the range were diluted and analyzed in duplicate.

### Statistical analysis

All statistical analyses were performed using SPSS software, version 22.0 (IBM Corp., Armonk, NY, USA). The normality of distribution for continuous variables was assessed using the Shapiro–Wilk test. Since most variables were not normally distributed according Shapiro-Wilk test, continuous variables are presented as median and interquartile range (Q1–Q3), and categorical variables as counts and percentages. Categorical variables were compared using the chi-square test. Changes in hematological, inflammatory, and neurotrophic parameters over time (Day 1, Day 4, and at discharge) were analyzed using the Friedman test for related samples due to the non-normal distribution of the data. For parameters with a significant overall Friedman test result, Wilcoxon signed**-**rank tests were performed as post-hoc pairwise comparisons to determine which time points differed significantly. For variables in which the overall Friedman test was not statistically significant, post-hoc Wilcoxon signed-rank tests were performed on an exploratory basis only and were not considered confirmatory. To control for multiple comparisons across biomarkers and timepoints, p-values were adjusted using the Benjamini–Hochberg false discovery rate (FDR) method.

Comparisons of neurotrophic factor levels (NGF and GDNF) at hospital admission in COVID-19 patients versus healthy controls were performed using the Mann–Whitney U test. A p-value < 0.05 was considered statistically significant.

A post hoc power analysis was performed using *G*Power version 3.1 to evaluate the adequacy of the sample size^15^. Assuming a large effect size (Cohen’s d = 0.8), α = 0.05, and two-tailed testing, the achieved statistical power (1–β) was 0.78, indicating sufficient power to detect large effects for the main comparisons.

## Results

A total of 30 patients diagnosed with COVID-19 and 37 healty volunteers were included in the study. Baseline demographic, hematological, inflammatory, and neurotrophic characteristics of COVID-19 patients and healthy controls are summarized in Table 1. There was no significant difference between the groups in terms of age or gender distribution (*p* = 0.655 and *p* = 0.945, respectively). While total white blood cell counts were comparable between COVID-19 patients and healthy controls (*p* = 0.440), platelet counts were significantly lower in the COVID-19 group at hospital admission (*p* < 0.001). Markers of systemic inflammation were markedly elevated in COVID-19 patients, with significantly higher erythrocyte sedimentation rate (ESR) and C-reactive protein (CRP) levels compared with healthy individuals (both *p* < 0.001). Notably, serum neurotrophic factor levels differed substantially between groups. Median NGF levels were significantly reduced in COVID-19 patients compared to healthy controls (*p* = 0.002), and a similar pattern was observed for GDNF, which was also significantly lower in the patient group (*p* < 0.001). These findings may indicate that acute COVID-19 infection is associated with pronounced inflammatory activation accompanied by a concurrent suppression of circulating neurotrophic factors at the time of hospital admission.

**Table 1:** Baseline characteristics of COVID-19 patients and healthy controls.

Parameter [Reference Ranges]	Healthy controls	COVID-19 patients	*p*-value
**Age (years)**	59 (46.5–68.0)	60.6 (44.5-75.25)	0.655
**Gender (Female/Male)**	22/15	19/11	0.945
**WBC (cells/µL)** [4000–10000 cells/ µL]	7080 (6005–7765)	6130 (4710–8200)	0.440
**Platelet (×10³/µL)** [150–450 × 10³/µL]	269 (232–294)	196 (152.5–232.5)	**< 0.001**
**ESR (mm/h)** [< 20 mm/h]	18 (16.6–22.0)	46 (34–81.5)	**< 0.001**
**CRP (mg/L)** [< 5 mg/L]	6 (3-9.5)	43.45 (15.69–94.96)	**< 0.001**
**NGF (ng/mL)**	48.12 (18.96–89.42)	22.1(15.5–29)	**0.002**
**GDNF (pg/mL)**	757.52 (442.43–1642.70)	341.5(243.95– 409.75)	**< 0.001**

WBC: White Blood Cell, ESR: Erythrocyte Sedimentation Rate, NGF: Nerve Growth Factor, GDNF: Glial-Derived Neurotrophic Factor p-value < 0.05 indicates a statistically significant difference. Values are presented as median (IQR: interquartile range). Between-group comparisons (COVID-19 patients vs. healthy controls) were performed using the Mann–Whitney U test for continuous variables and the χ² test for categorical variables (gender).

**Fig. 1:**
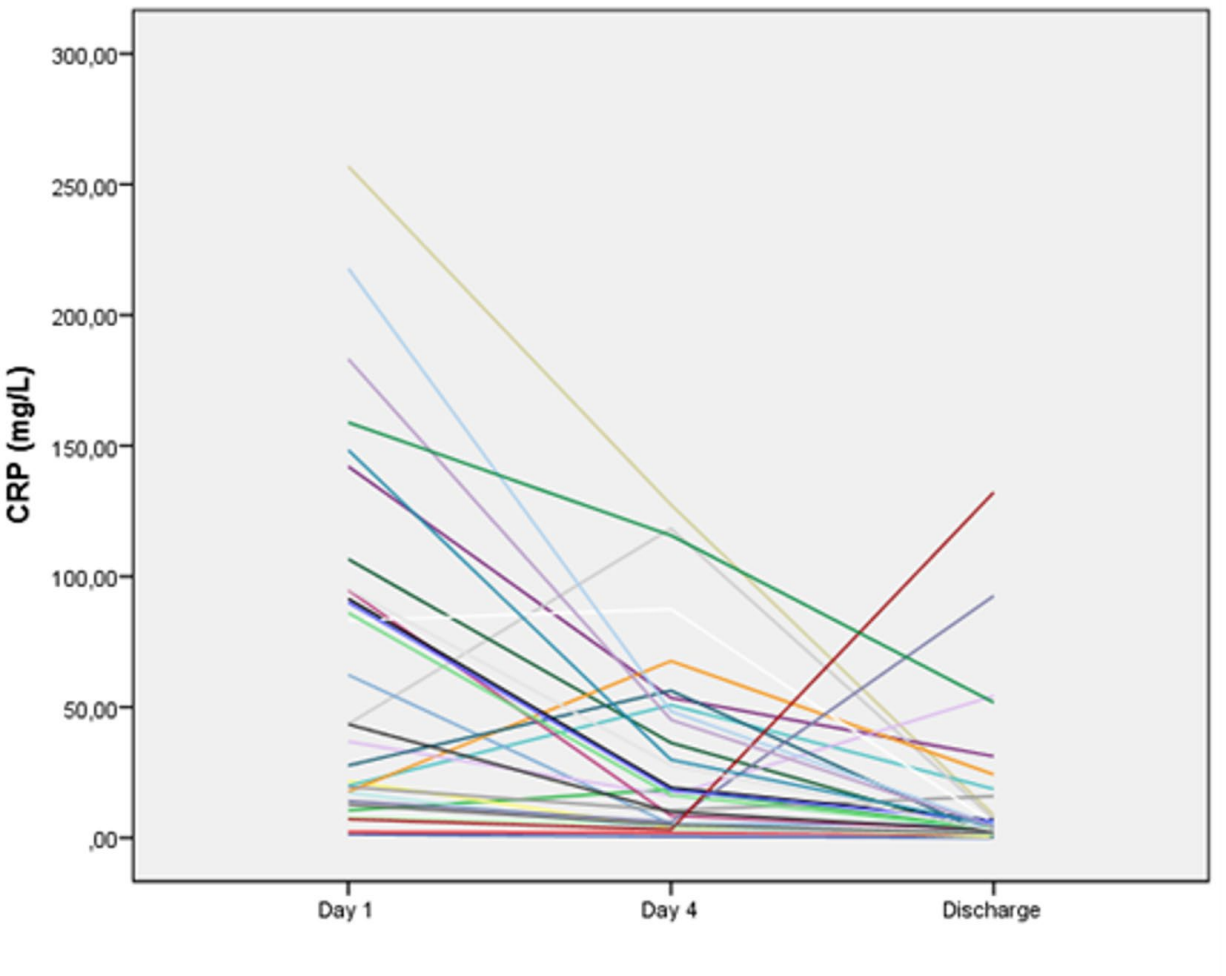
Individual trajectories of C-reactive protein (CRP) levels in hospitalized COVID-19 patients measured at Day 1, Day 4, and at discharge. Each line represents one participant, illustrating inter-individual variability during recovery.

Longitudinal changes in hematological, inflammatory, and neurotrophic parameters of COVID-19 patients during hospitalization are presented in Table 2. At baseline (Day 1), patients exhibited elevated inflammatory markers, including CRP (Fig. 1), ESR, ferritin, fibrinogen, and D-dimer, along with alterations in leukocyte subpopulations. During follow-up, a gradual decline in several systemic inflammatory markers was observed, particularly CRP and fibrinogen, indicating partial resolution of the acute inflammatory response. In contrast, ferritin and D-dimer levels did not show statistically significant longitudinal changes.

**Table 2:** Changes in all parameters over time.

Parameter [Reference Ranges ]	Day 1	Day 4	Discharge	*p* (Friedman)	Wilcoxon Post-hoc (*p*)
**WBC (cells/µL)** [4000–10000 cells/ µL]	6130 (4710–8200)	9850 (7415–11955)	9640 (7165–12675)	**< 0.001**	D1 vs. D4: <0.001* D1 vs. Dis: <0.001* D4 vs. Dis: 0.781
**Lymphocytes (cells/µL)** [1000–4800 cells/ µL]	1040 (795–1475)	840 (535–1145)	800 (530–1420)	0.107	**D1 vs. D4: 0.016** D1 vs. Dis: 0.349 D4 vs. Dis: 0.213
**Platelets (×10³/µL)** [150–450 × 10³/µL]	196 (152.5–232.5)	241 (214–313.5)	287 (230.5–359.5)	**< 0.001**	D1 vs. D4: <0.001 * D1 vs. Dis: <0.001* D4 vs. Dis: 0.010*
**Neutrophils (cells/µL)** [2000–7000 cells/ µL]	3690 (3165–6110)	7690 (6055–10715)	7830 (5565–10625)	**< 0.001**	D1 vs. D4: <0.001* D1 vs. Dis: <0.001* D4 vs. Dis: 0.805
**LDH (U/L)** [140–280 U/L]	295 (257–364.5)	259 (212–340)	256 (212.5–293)	**0.007**	D1 vs. D4: 0.015* D1 vs. Dis: 0.015* D4 vs. Dis: 0.382
**Fibrinogen (mg/dL)** [200–400 mg/dL]	500 (445.5–609)	454 (380.5–513)	336 (297–429.5)	**< 0.001**	D1 vs. D4: 0.001* D1 vs. Dis: <0.001* D4 vs. Dis: <0.001*
**Ferritin (ng/mL)** [Female:10–150 ng/mL] [Male: 20–300 ng/mL]	191.2 (90–434.25)	197.4 (100.1–440.95)	244.2 (108.3–612.7)	0.177	D1 vs. D4: >0.05 D1 vs. Dis: >0.05 D4 vs. Dis: >0.05
**ESR (mm/h)** [< 20 mm/h]	46 (34–81.5)	49 (28–76.5)	34 (21.5–57.5)	**0.018**	D1 vs. D4: 0.399 D1 vs. Dis: 0.024* D4 vs. Dis: 0.018*
**CRP (mg/L)** [< 5 mg/L]	43.45 (15.69–94.96)	18.3 (5.1–49.63)	4.84 (1.96–12.305)	**< 0.001**	D1 vs. D4: 0.002* D1 vs. Dis: <0.001* D4 vs. Dis: 0.003*
**D-dimer (mg/L)** [< 0.5 mg/L]	0.52 (0.325–1.195)	0.31 (0.17–0.57)	0.38 (0.22–0.885)	0.103	**D1 vs. D4: 0.017** D1 vs. Dis: 0.269 D4 vs. Dis: 0.585
**GDNF (pg/mL)**	341.5 (243.95–409.75)	314.9 (237.7–381.4)	322 (271.4–435.5)	0.503	D1 vs. D4: 0.497 D1 vs. Dis: 0.914 D4 vs. Dis: 0.838
**NGF (ng/mL)**	22.1 (15.5–29)	17.6 (11.85–24.05)	19.2 (13.8–26.2)	0.114	**D1 vs. D4: 0.014** D1 vs. Dis: 0.336 D4 vs. Dis: 0.559

WBC: White Blood Cell, ESR: Erythrocyte Sedimentation Rate, NGF: Nerve Growth Factor, GDNF: Glial-Derived Neurotrophic Factor. p-value < 0.05 indicates a statistically significant difference. Values are presented as median (IQR: interquartile range). Post-hoc comparisons were performed using the Wilcoxon signed-rank test, with p-values adjusted for multiple testing using the Benjamini–Hochberg false discovery rate (FDR). * indicates comparisons remaining significant after FDR correction (q ≤ 0.05).

To further characterize inflammatory recovery at the individual level, we analyzed the proportion of patients remaining above clinical reference thresholds at discharge. While group-level median values demonstrated an overall downward trend, inter-individual variability was observed. At discharge, ESR remained elevated (> 20 mm/h) in 76.7% of patients, ferritin (> 300 ng/mL for men, > 150 ng/mL for women) in 56.7%, and CRP (> 5 mg/L) and WBC (> 10000 cells**/**µL) in 46.7% of the cohort. D-dimer (> 0.5 mg/L) and fibrinogen (> 400 mg/dL) levels were above reference ranges in 43.3% and 36.7% of patients, respectively.

**Fig. 2:**
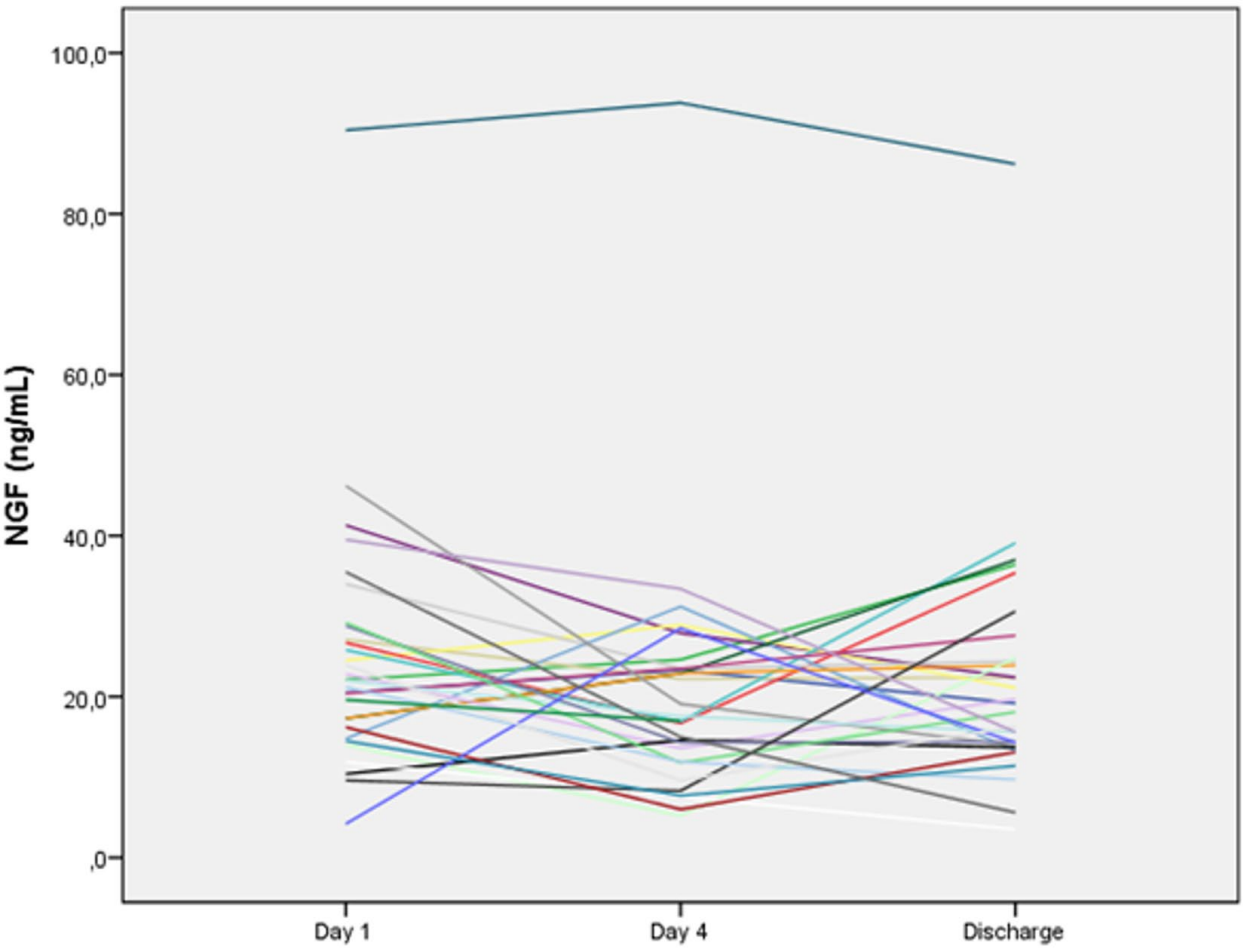
Longitudinal changes in serum nerve growth factor (NGF) levels in COVID-19 patients during hospitalization. Individual participant trajectories are shown to highlight within-subject dynamics over time.

**Fig. 3:**
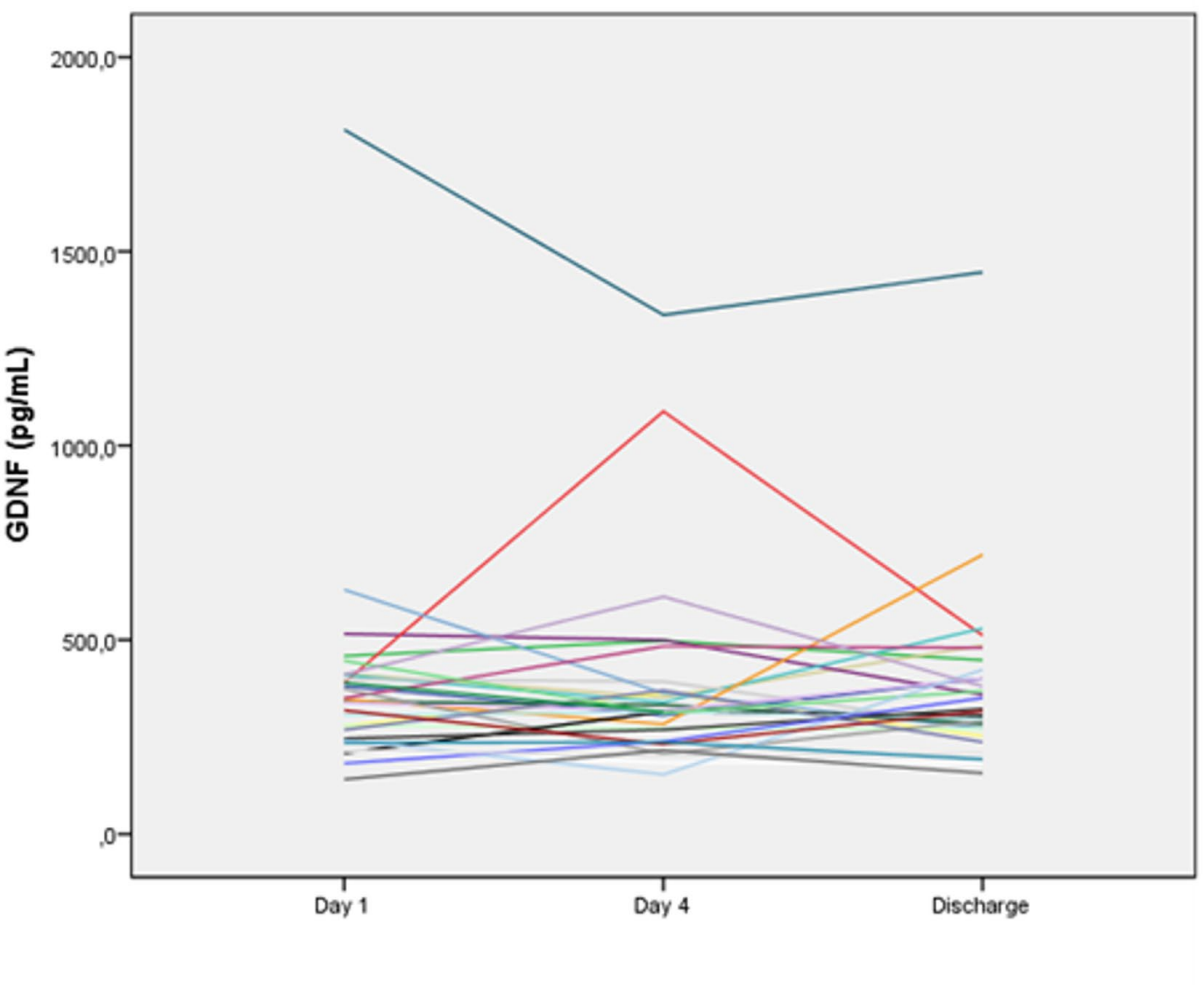
Serum glial cell line–derived neurotrophic factor (GDNF) levels across hospitalization in COVID-19 patients. Individual trajectories demonstrate the relative stability of GDNF levels despite clinical recovery.

With respect to neurotrophic factors, both NGF and GDNF levels demonstrated a biphasic pattern during follow-up (Table 2; Figs. 2 and 3). From Day 1 to Day 4, a decrease in serum levels was observed for both neurotrophic factors, followed by a subsequent increase at later time points. Although the overall Friedman test did not indicate a statistically significant longitudinal change in NGF levels, an exploratory pairwise comparison suggested a transient numerical decrease between Day 1 and Day 4.

## Discussion

This study investigated the trajectory of inflammatory (CRP, ESR, fibrinogen, ferritin, D-dimer), hematological (white blood cells, neutrophils, platelets, lymphocytes), and neurotrophic (NGF, GDNF) markers in patients hospitalized with a diagnosis of COVID-19 and compared them with a control group of healthy individuals. The findings revealed that NGF and GDNF levels were significantly lower in COVID-19 patients compared to healthy controls. Although NGF levels were relatively high in the early period, they exhibited a decrease by the fourth day, followed by a partial recovery at discharge. While the overall change in NGF across hospitalization did not reach statistical significance in global testing, the early decrease may indicates that NGF could be sensitive to acute inflammatory activation. In contrast, GDNF levels remained stable throughout the disease course. These results suggest that NGF may be a dynamic indicator of the acute immune response, while GDNF might be associated with more stable, chronic processes.

The significantly lower levels of NGF found in COVID-19 patients compared to healthy individuals indicate the suppressive effect of systemic inflammation on the neuroimmune axis. Previously, Petrella et al. (2022) reported that NGF levels remained low even during the recovery period in adolescents with long COVID.^8^Similarly, Özgen et al. (2025) stated that NGF levels were severely reduced in Behçet’s disease patients, noting that this is a direct reflection of the inflammatory response.^3^NGF regulates cytokine release through its interactions with immune cells such as mast cells and monocytes; however, under excessive inflammatory load, this mechanism can be suppressed.^16^Therefore, the suppression of NGF expression in SARS-CoV-2 infection may pave the way for both uncontrolled immune cell activity and the emergence of neurological sequelae. However, these apparent early fluctuations in NGF levels should be interpreted with caution, as the overall longitudinal analysis did not reach statistical significance and the observed pairwise differences were exploratory in nature.

In our study, GDNF levels in COVID-19 patients were also significantly lower than in healthy individuals. This finding indicates that GDNF levels may be reduced during systemic inflammation, potentially reflecting indirect effects of the immune response rather than direct viral suppression. Chen et al. (2022) reported that in inflammatory bowel disease^17^, GDNF levels did not exhibit a consistent increase during active inflammation but rather showed variability. Although Türkeri et al. (2023) did not find a significant difference in GDNF levels between COVID-19 patients and healthy controls, the low values obtained in our study suggest that SARS-CoV-2 may exert a suppressive effect by increasing the inhibition of peripheral GDNF production or utilization.^18^Furthermore, Özgen et al. (2025) supported this finding by demonstrating that both NGF and GDNF were severely reduced in Behçet’s disease patients.^3^These findings suggest that GDNF may be less responsive to acute-phase changes and could be involved in longer-term neuroimmune modulation, although further studies are required to confirm this.

In the early phase of COVID-19, NGF levels decreased modestly, while CRP levels increased significantly—a pattern indicating opposite trajectories during acute inflammation. CRP rises rapidly as part of the acute-phase response, whereas NGF appears to be suppressed as inflammation intensifies.^12^This dynamic change in NGF suggests that it reflects immune activation in the early period and subsequently recedes with the resolution of inflammation. In contrast, GDNF levels remained stable throughout the illness, showing no significant fluctuation. This may indicate that GDNF is less sensitive to the rapid changes of the acute immune response, but its low baseline levels may signal chronic neuroimmune suppression. Consequently, while NGF emerges as a dynamic biomarker reflecting the acute inflammatory course of COVID-19, GDNF may be more indicative of long-term immune regulation processes. Notably, despite the overall downward trend in inflammatory markers at the group level, a proportion of patients were discharged with values remaining above reference ranges, highlighting inter-individual variability in biochemical recovery.

Our findings contribute to a broader understanding of the neuroimmune axis in COVID-19 and potentially other severe viral infections. The early decrease in NGF, occurring alongside reductions in acute-phase reactants such as CRP and fibrinogen, positions NGF as a potential marker of early inflammatory response and recovery. The overall improvement in CRP and fibrinogen aligns with the modest rebound in NGF observed at discharge may indicate a transition from hyperinflammation to resolution, offering clinicians a potential tool to gauge the effectiveness of anti-inflammatory treatments and predict clinical course.

In contrast, the levels of GDNF, which showed no significant fluctuation during hospitalization yet remained markedly lower than in healthy controls, suggest its role may be more associated with long-term neuroimmune dysregulation than acute phase monitoring. Persistently low GDNF levels observed during acute illness may warrant investigation in relation to long COVID symptoms, given prior evidence of neuroimmune involvement in post-viral syndromes.^8,18,19^Future studies should investigate whether GDNF levels remain suppressed in patients with long COVID and if they correlate with specific neurological symptoms.

From a therapeutic perspective, these neurotrophins may represent potential targets for future investigation.Strategies aimed at boosting NGF signaling in the early phase might help modulate excessive immune activation, whereas interventions designed to normalize GDNF pathways could be beneficial in mitigating long-term neuropsychiatric complications.^20,21^However, any therapeutic approach must be considered cautiously due to the dual and context-dependent roles of neurotrophins in both promoting and resolving inflammation.^22^.

This study has several limitations. The relatively small sample size may limit statistical power and generalizability. The cohort was predominantly older, which could influence neurotrophin levels due to age-related changes in immune and neural function. Neurotrophin measurements were restricted to serum levels, which may not accurately reflect their concentrations or activity within the central nervous system or local inflammatory sites. Clinical outcome data (e.g., ICU admission, oxygen requirement, or length of hospitalization), treatment information, and standardized severity scores were not systematically recorded, which may have influenced inflammatory and neurotrophin levels and limits conclusions regarding their clinical relevance. Additionally, the timing of discharge sampling varied across patients (approximately Day 7 to Day 14), and treating discharge as a single timepoint may have obscured inter-individual differences in biomarker trajectories and complicated the interpretation of longitudinal trends. In addition, the lack of post-discharge follow-up prevents assessment of long-term changes in NGF and GDNF or their relationship to post-COVID manifestations. Future studies with larger, more age-diverse populations and comprehensive clinical data are needed to validate and extend these findings.

In conclusion, this longitudinal study provides novel evidence of the distinct dynamics of two key neurotrophins, NGF and GDNF, during acute COVID-19. NGF levels showed a modest early decrease in the early phase of hospitalization, paralleling changes in conventional inflammatory markers, suggesting its role as a dynamic biomarker of acute neuroimmune activation and subsequent resolution. In stark contrast, GDNF levels remained stable and significantly suppressed throughout the disease course, indicating its potential involvement in chronic neuroimmune processes rather than acute fluctuations. Given the limited data on GDNF dynamics in viral infections, our findings should be interpreted with caution and viewed as hypothesis-generating rather than confirmatory.

These findings underscore the intricate interplay between the nervous and immune systems in COVID-19 and highlight NGF and GDNF as key players in this crosstalk. Further research is warranted to explore their utility as biomarkers for disease monitoring, prognostic tools, and potential therapeutic targets, not only in COVID-19 but also in other conditions characterized by significant neuroimmune dysregulation.

## Data Availability

The datasets generated and/or analyzed during the current study are available from the corresponding author on reasonable request.
